# Can Prenatal Diagnosis of Total Anomalous Pulmonary Venous Return (TAPVR) Using Routine Fetal Ultrasound be Improved? A Case-Control Study

**DOI:** 10.1155/2022/7141866

**Published:** 2022-12-31

**Authors:** Jack Heard, Reeni Soni, Katarina Nikel, Chelsea Day, Christy Pylypjuk

**Affiliations:** ^1^Department of Obstetrics, Gynaecology and Reproductive Sciences, University of Manitoba, Winnipeg, Manitoba, Canada; ^2^Department of Pediatrics and Child Health, University of Manitoba, Winnipeg, Manitoba, Canada; ^3^Department of Obstetrics, Gynaecology and Reproductive Sciences, University of Saskatchewan, Saskatoon, Saskatchewan, Canada; ^4^Children's Hospital Research Institute of Manitoba, Winnipeg, Manitoba, Canada

## Abstract

**Objectives:**

To determine the most common fetal ultrasound markers of total anomalous pulmonary venous return (TAPVR) during mid-trimester ultrasound using standardly obtained images and evaluate the performance of diagnostic algorithms for improving prenatal diagnosis.

**Methods:**

This was a matched case-control study at a regional referral centre (2005 to 2019). Cases of TAPVR were matched to controls 1 : 4 by date of birth and biologic sex. Postprocessing review of stored fetal ultrasound images was performed by two blinded and independent observers in a standardized fashion using nine sonographic markers: (i) left/right heart disproportion; (ii) abnormal distribution of great vessels; (iii) pulmonary vein entry into the left atrium (LA); (iv) confluence behind the LA; (v) abnormal coronary sinus; (vi) absence of the Coumadin ridge; (vii) aortic diameter; (viii) distance between LA and aorta; and (ix) post-LA space index >1.27. Descriptive and inferential statistics were used to present results and compare cases and controls. Diagnostic algorithms were compared by sensitivity/specificity.

**Results:**

21 cases of isolated TAPVR were matched to 84 controls (*n* = 105). The most common ultrasound marker of TAPVR was absence of pulmonary vein entry into the LA (42.9%), followed by abnormal Coumadin ridge (38.1%). Cases of TAPVR had significantly larger post-LA spaces than controls (*p* < 0.0001) and wider aortic diameters (*p*=0.006). A diagnostic algorithm stratifying on absence of pulmonary veins followed by an abnormal Coumadin ridge, can correctly identify cases of TAPVR with high specificity (90.5%) and moderate sensitivity (61.9%). Conversely, a diagnostic algorithm using the presence of any 3 abnormal markers had improved specificity (94.1%) but poorer sensitivity (23.8%).

**Conclusions:**

Using standardly obtained images from routine fetal ultrasound, improved prenatal detection of isolated TAPVR is possible. A standardized diagnostic approach can be highly specific for fetal TAPVR, however, algorithms that are sufficiently sensitive for screening in the general population are still needed.

## 1. Introduction

Total anomalous pulmonary venous return (TAPVR) is a cardiac malformation where the four pulmonary veins fail to connect normally to the left atrium [1]. This represents 0.5–2% of all cardiac anomalies detected in newborns, with an incidence of 6–12 cases per 100,000 live births [1, 2]. While a less common type of cardiac anomaly, TAPVR is associated with acute cardiorespiratory decompensation after birth and is the fifth leading cause of critical heart disease in newborns [1, 3]. For this reason, accurate identification of fetuses affected by TAPVR is essential for improving postnatal outcomes. However, prenatal detection remains challenging, with only 2% to 10% of all cases being diagnosed before birth [1, 4]. Prenatal diagnosis of TAPVR is particularly difficult when it is isolated and in the absence of other cardiac anomalies [1, 4]. Failure to diagnose TAPVR prenatally means that affected newborns may be delivered at rural/community hospitals or other low-risk settings, without the specialized resources to manage complex cardiac lesions, and risks heightened morbidity and mortality [1, 5].

TAPVR is included within the spectrum of bronchopulmonary malformations that comprise “pulmonary sequestration” [6, 7]. There are two embryologic theories on the process of pulmonary veins (PV) establishing a normal connection to the left atrium (LA). The first theory describes a budding from the LA dorsal wall that grows into the developing lung parenchyma to connect to the developing PV directly [8]. The second theory suggests that the early lung tissue, the pulmonary bud, is connected to the splanchnic plexus of blood vessels that drain into the systemic vascular system [8]. By 4 weeks gestation, the primordial PV buds off the LA and will connect with the pulmonary portion of this splanchnic plexus [8]. Over time, this pulmonary circulation separates from the systemic vasculature, leaving an isolated pulmonary system [8]. This latter theory better explains TAPVR, where the primordial PV fails to establish connection to the pulmonary circulation, thus leaving it connected to systemic veins [8].

With advances in obstetrical ultrasound technology and the implementation of 3D/4D fetal echocardiography, prenatal diagnosis of cardiac anomalies is improving [5]. The role of magnetic resonance imaging (MRI) in prenatal diagnosis shows promise as well, with technological advantages over ultrasound including larger fields of view, less dependence on fetal position, and better resolution in larger patients [9]. Fetal cardiac MRI is currently under investigation for prenatal diagnosis of cardiac lesions, though challenges still exist due to the small heart size, fast fetal heat rate, unpredictable fetal movements, and maternal respirations [9]. With TAPVR being a primary cardiac lesion, prenatal diagnosis suffers from these limitations [9]. However, not all prenatal patients have access to specialized fetal echocardiography or MRI services that are typically only available in larger centres [5]. Additionally, a referral for fetal echocardiography and advanced imaging requires an initial suspicion that a cardiac anomaly exists. Because less attention is paid specifically to pulmonary venous return on the four standard views of the fetal heart during routine mid-trimester obstetrical ultrasound—the four chamber view, great vessel view, right ventricular outflow tract, and left ventricular outflow tract—many fetuses affected by isolated TAPVR may never reach the expert eye of a pediatric cardiologist antenatally [10].

Fetal ultrasound markers of TAPVR have been proposed to improve prenatal diagnosis [4, 5]. While most of these markers require additional imaging techniques, several can be seen from the four standard cardiac views described above. The purpose of this study was to develop a diagnostic algorithm for these markers that could be implemented using standard images from mid-trimester obstetrical ultrasound (or “routine anatomy scan”) to help improve prenatal detection of TAPVR. By improving prenatal detection, this will allow for the triage of care to sites where appropriate management and counselling can be provided for these infants and their families.

## 2. Materials and Methods

This was a retrospective, case-control study from a tertiary-level hospital in Winnipeg, Canada (January 1, 2005, to December 31, 2019). Cases of TAPVR were identified in a pediatric cardiology clinic repository. This clinic serves as a regional referral site for all pediatric cardiology consults in a population of 1.3 million individuals with a demographic composed of rural, urban, and northern/remote communities [11]. Cases included both fetuses diagnosed with TAPVR antenatally and newborns diagnosed postnatally. All diagnoses of TAPVR were confirmed by postnatal echocardiography. Healthy control newborns were identified using hospital delivery record books and matched to cases of TAPVR in a 4 : 1 fashion by calendar date of birth and biologic sex. We excluded newborns with multiple anomalies, prelabour stillbirth, planned termination, or postnatal palliation, as well as those with missing ultrasound images. There were two main outcomes of interest: (i) the frequency of TAPVR markers amongst fetuses at the time of mid-trimester ultrasound (18–23 weeks' gestation) using standardly obtained images; and (ii) performance of diagnostic algorithms for the identification of fetuses with TAPVR.

**Figure 1:**
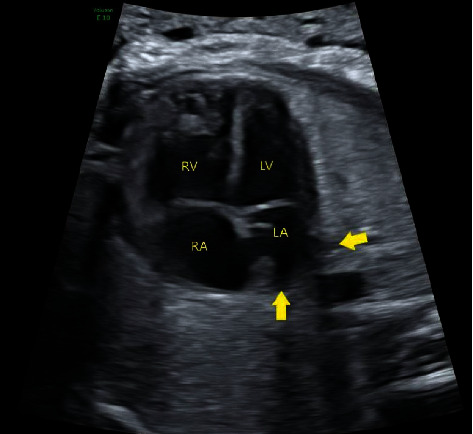
Standard 2D greyscale ultrasound image of the 4-chamber view of the fetal heart with pulmonary vein entry into the left atrium (LA) indicated by yellow arrows (legend: LA = left atrium, RA = right atrium, LV = left ventricle, and RV = right ventricle.).

**Figure 2:**
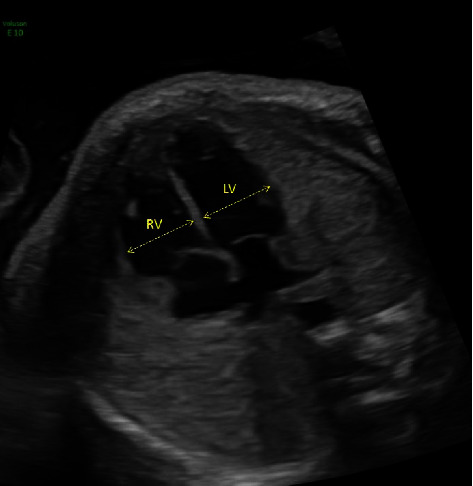
Standard 2D greyscale ultrasound image of the 4-chamber view of the fetal heart, illustrating balanced right (RV) and left ventricles (LV) (legend: RV = right ventricle and LV = left ventricle.).

**Figure 3:**
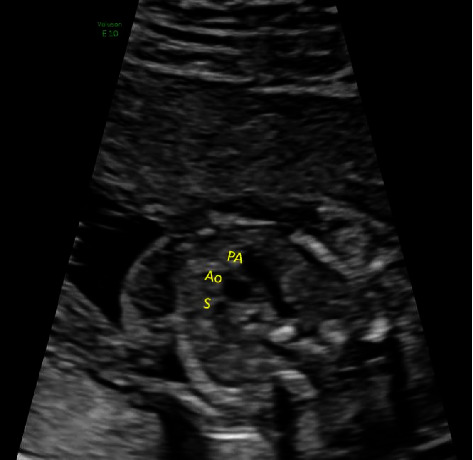
2D greyscale ultrasound image of a transverse plane of the fetal thorax at the level of the great vessels, illustrating normal spacing between the pulmonary artery (PA), aorta (Ao), and superior vena cava (S) (legend: PA = pulmonary artery, Ao = aorta, and S = superior vena cava.).

**Figure 4:**
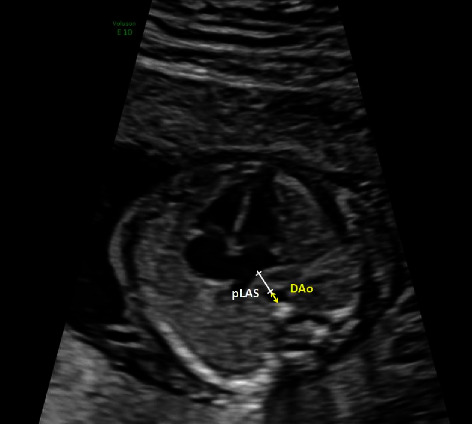
Standard 2D greyscale ultrasound image of the 4-chamber view of the fetal heart with callipers (white) measuring the post-LA space (pLAS) and yellow callipers measuring the diameter of the descending aorta (DAo). (legend: pLAS = postleft atrial space, and DAo = descending aorta.).

All obstetrical ultrasound images performed in the region since 2005 are stored centrally and available for electronic viewing. The post-processing review of stored obstetrical ultrasound images from routine mid-trimester scans was performed by two blinded and independent observers. Using a standardized approach as previously described, the following markers were evaluated: (i) presence of a confluence behind the left atrium (LA); (ii) pulmonary vein entry into the LA (Figure 1); (iii) presence of an abnormal coronary sinus; (iv) presence of an abnormal Coumadin ridge; (v) evidence of left-to-right heart disproportion (Figure 2); (vi) abnormal spacing of the great vessels on the standard three vessel view (Figure 3); (vii) distance between the aorta and LA (“post-LA space”) (Figure 4); (viii) aortic diameter; and (ix) index of post-LA space-to-aortic diameter (“post- LA index”) (Figure 4) [4]. Primarily, these ultrasound findings were evaluated as binary markers (present or absent). Continuous measurements were presented both as means with standard deviations and then dichotomized using either the mean or 95^th^ percentiles as cut-offs. In addition, the post-LA index was evaluated using a cut-off of 1.27, as previously described in the literature [12].

Statistical analysis was performed using Stata v.14.2 (Stata Corp, College Station, TX, USA) software. Descriptive statistics were used to present the characteristics of the participants. Continuous variables were described as means with standard deviations (SD), and dichotomous/categorical variables were described as proportions (in %). Chi-square and paired *t*-tests were used to compare findings between groups. Using the frequencies of individual markers and combined markers, diagnostic algorithms were developed and evaluated by calculating the sensitivity and specificity for each. A Bland-Altman plot was used to assess interobserver reliability. Institutional ethics approval was obtained from the University of Manitoba Health Research Ethics Board. Individual patient consents were obtained for the use of fetal ultrasound images.

## 3. Results

**Figure 5:**
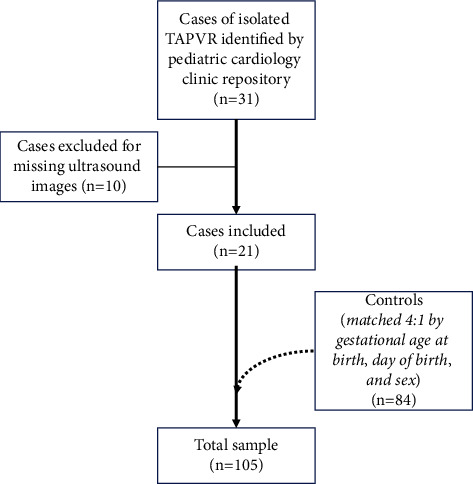
Flow diagram of study participants.

From the clinical repository, 31 cases of isolated TAPVR were identified during the study period (Figure 5). 10 cases were excluded due to missing ultrasound images. These were matched to 84 controls with stored mid-trimester obstetrical ultrasound images, resulting in a total study sample of 105 (Figure 1). The gestational age at the time of mid-trimester ultrasound was different between cases and controls, as those with TAPVR tended to have later scans (22 versus 21 weeks' gestation; *p*=0.028). It was possible to evaluate almost all markers using the standard cardiac views between 18 to 22 weeks' gestation. Specifically, for only 1 case of TAPVR was it not technically possible to evaluate all ultrasound markers on standard views. There was good interobserver reliability. For measurement of the post-LA space, the Bland–Altman plot with 95% limits of agreement was −0.05 to 0.04 cm and confirmed the absence of systemic error; for measurement of the aortic diameter, the 95% limits of agreement −0.06 to 0.03 cm.

The most common ultrasound marker in cases of TAPVR was the absence of visualization of pulmonary vein entry into the left atrium (42.9%), followed by an abnormal Coumadin ridge (38.1%) and a post-LA index >1.27 (28.6%) (Table 1). Amongst controls, the distribution of abnormal markers was similar, although the relative frequencies were lower (Table 1). Interestingly, for 44% of healthy fetuses there was also nonvisualization of pulmonary veins into the LA on standardly obtained, 2D grayscale views (*p* = 0.936). Cases of TAPVR also had significantly larger post-LA spaces (0.41 cm (SD 0.14)) than controls (0.31 cm (SD 0.09); *p* < 0.0001), and wider aortic diameters (0.38 cm (SD 0.11) versus 0.32 cm (SD 0.08); *p* = 0.006) (Table 1). Only one-third of fetuses with TAPVR (28.6%) had a post-LAindex measuring above the 1.27 cut-off previously published [12].

**Figure 6:**
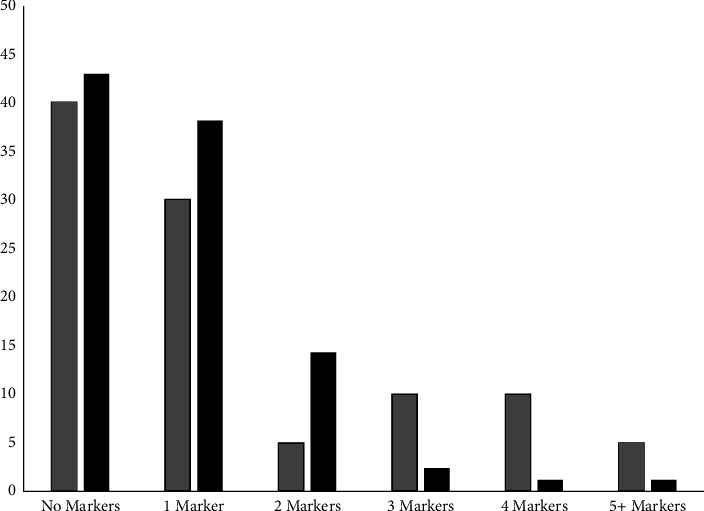
Frequency (in %) of ultrasound markers of TAPVR between cases (black) and controls (light grey).

When evaluating for the presence of any abnormal markers, there was no significant difference between cases and controls when any 1, 2, or 3 markers in combination were used for diagnosis (Figure 6). Fetuses with TAPVR were more likely to have 4 abnormal markers compared to controls (*p*=0.034) (Figure 6). When the reference cut-off for the post-LA space index of 1.27 was used to define any 3 or more markers, there were increased odds of TAPVR of 6.25 (95% CI 1.5–25.9); when the 95^th^ percentile from our data set was used instead, the odds of TAPVR were slightly lower (OR 4.94 (95% CI 1.3–19.1); *p*=0.021). There was no difference between cases and controls with respect to the presence of any 5 or more markers, but the absolute numbers were small (Figure 6). After evaluating the relative frequencies and significant differences of individual markers between groups, two types of diagnostic algorithms were developed. The first type was based exclusively on the total number of any markers. The second type utilized most prevalent individual markers with stratification by next common or the next most significant markers.

Using a diagnostic algorithm based simply on the presence of any 3 abnormal markers, cases of TAPVR can be detected with 97.6% specificity (95% CI 91.7–99.7%) but only 10% sensitivity (95% CI 1.2–31.7%) (Table 2). When any 4 markers are used, the sensitivity remains unchanged (10%; 95% CI 1.2–31.7%) and the specificity improves to 98.8% (95% CI 93.5–99.9%) (Table 2). With a diagnostic algorithm developed by stepwise evaluation of the most common markers seen—stratifying first by lack of pulmonary vein entry into the LA followed by the presence of an abnormal Coumadin ridge—cases of TAPVR can be accurately identified with high specificity (90.5%) and moderate sensitivity (61.9%) (Table 2). Alternatively, an algorithm starting with pulmonary vein entry into the left atrium followed by the presence of any 3 or more markers has better specificity (94.1%) but lower sensitivity (23.8%) (Table 2).

## 4. Discussion

Improving the prenatal diagnosis of TAPVR is possible. With a systematic approach to evaluating markers of TAPVR, affected fetuses can be accurately identified at the time of mid-trimester ultrasound using standardly obtained cardiac views without incurring any additional costs or hospital resources. Of all markers under investigation, there was the most marked difference in post-LA space and aortic diameter between cases and controls; however, their evaluation does involve the additional application of measurement callipers to standard images. While not routinely imaged, pulmonary vein entry was incidentally seen on stored, 2D grayscale images of the four-chamber heart view in fetuses from both groups. Not surprisingly, lack of pulmonary vein entry was the most common marker identified in fetuses with TAPVR. However, this marker was also present in almost 40% of controls. Interestingly, the relative distribution of abnormal markers between cases and controls was fairly similar. Continuous measurements were reported as means with standard deviations, but in order to create the most practical algorithm that could easily be implemented in general practice, the algorithms developed and evaluated only utilized dichotomous markers. While specificity was high for all algorithms we evaluated (90–98%), sensitivity was only low to moderate (10–62%). Because the specificity between algorithms was similar, the algorithm based simply on the presence/absence of any 3 or 4 markers may be practically easier to implement in the clinical setting; however, the sensitivity was poorest for algorithms utilizing any markers versus a step-wise approach. Our study shows promise for these markers in improving the specificity of the fetal diagnosis of TAPVR; however, additional work is needed to improve the level of sensitivity required for screening at the population-level.

With advances in fetal echocardiography techniques, there exists a heightened capacity for detecting more subtle cardiac lesions before birth. However, there is evidence that antenatal detection of congenital heart disease remains reduced when imaging occurs in smaller settings with a lower clinical volume [13, 14]. From a few small studies that have been published, visualization of pulmonary venous flow into the LA by color Doppler seems to be the most reproducible marker of TAPVR and thus should be considered for implementation into routine screening [4, 5]. However, this view involves advanced training, particularly given the existing challenges in some centers with acquiring the standard outflow tract views in sufficient detail [15]. The original paper from Olsen et al. included only 10 cases of TAPVR and a total study sample of 19, and sensitivity/specificity were not reported, and thus, we are unable to draw direct comparisons with our study. Another study using 3D fetal techniques also identified pulmonary venous flow into the LA as the best markers of TAPVR [5]. Measurement of the post-LA space does require some additional training to ensure reproducibility and reliability. Our study showed good interobserver reliability of this marker, but the absolute mean difference was small. Kawazu et al. reported larger differences in post-LA spaces between groups by the early third trimester, but there were only 8 cases of TAPVR included in their analysis [12]. Their small sample size and later gestational age of scans may explain why the post-LA space index was above the 1.27 cut-off in only one-third of our fetuses with TAPVR [12]. Because third-trimester ultrasounds are not routinely performed, reference ranges of these markers are needed in mid-pregnancy to coincide with the timing of the routine anatomy scan. The majority of cardiac anomalies occur in low-risk pregnancies without obvious risk factors, and 2D grayscale images on routine obstetrical ultrasound are the primary mechanism for identification [14]. As access to tertiary- (or quaternary-) level fetal echocardiography or MRI remains limited in many communities, implementation of a diagnostic algorithm from standardly collected views at 18–22 weeks' gestation could be transformative in prenatal diagnosis of TAPVR.

This need to improve prenatal diagnosis of TAPVR exists irrespective of its relative rarity in the general population. From our study, we see that prenatal diagnosis of TAPVR is feasible during routine obstetrical ultrasound. Our results also serve to improve awareness of subtle markers of TAPVR that are present during routine obstetrical ultrasound already by 18–22 weeks' gestation. Because the specificity of our diagnostic algorithms is high, we suggest that all cases with nonspecific “abnormal heart” findings on mid-trimester ultrasound have routine images evaluated for the presence/absence of markers of TAPVR using a standardized approach. Such a review can be performed without a need for additional imaging techniques or resources, particularly for those families without healthcare insurance or availability of fetal echocardiography in their home communities. We know that poorer outcomes exist for newborns with congenital heart disease born outside of a tertiary care center [16–18]. Infant mortality is high in TAPVR (up to 23%) even when birth occurs at centres with cardiac surgery services, which further underscores the need for improvements in postnatal care [1, 4]. In addition to better coordination of multidisciplinary care, accurate prenatal diagnosis with TAPVR allows time for facilitation of antenatal genetic testing and better counseling/preparation for families. At a minimum, heightened awareness comes with better chances for prenatal diagnosis of this elusive fetal anomaly.

Future research is still needed to develop highly sensitive diagnostic screening algorithms for improved detection of TAPVR in the general population. Inclusion of continuous ultrasound measurements in the risk-prediction modelling may improve the sensitivity/specificity of these algorithms, but that was beyond the scope of this project. However, the practicality of implementing more complex algorithms in general practice remains a concern. In addition, a better understanding of the specific measurement cut-offs of the post-LA space that correspond to heightened risk of TAPVR at mid-pregnancy would further improve the practical application of these diagnostic algorithms. Sex-specific differences in fetal ultrasound features—beyond estimated weight—remain understudied and warrant future consideration. Lastly, validation studies are needed to fully evaluate the performance of these algorithms in clinical practice.

Our study has several important strengths. This project highlights how systematic evaluation of specific markers at mid-pregnancy could improve prenatal diagnosis of TAPVR in the absence of other cardiac anomalies. Because case selection was achieved using a specialized clinic repository which serves as the central intake for all fetuses and children with suspected cardiac anomalies in the region, the cases enrolled in our study should reflect the background population from where they were sampled. The feasibility and utility of these markers are attested to by the strong interobserver reliability. Additionally, we were able to evaluate almost all markers using standardly obtained images at 18–22 weeks of gestational age. However, given the rarity of TAPVR, our sample size was small despite the 15-year study period, although we note that it is larger than many others published about TAPVR. Another inherent limitation of case-control studies is the risk of selection bias in sampling, although this risk should be minimized by using the regional repository. Stillbirths with undiagnosed or unconfirmed TAPVR would also not have been captured by our sampling strategy. Because biologic sex was used as a matching variable, we are unable to evaluate its independent influence on the presence/absence of these cardiac markers of TAPVR, which should be the focus of future study.

## 5. Conclusions

While TAPVR continues to be a diagnostic challenge in the antenatal period, prenatal diagnosis can be improved. By utilizing a standardized approach to the evaluation of unique markers during routine, mid-trimester views of the fetal heart, cases of TAPVR can be detected with high specificity but only low to moderate sensitivity. Future studies are still needed to improve prenatal screening of TAPVR in the general population.

## Figures and Tables

**Table 1:** Differences in mid-trimester ultrasound characteristics between cases and controls, expressed as frequencies (in %) or means (with standard deviations).

Ultrasound marker	Cases (*n* = 21)	Controls (*n* = 84)	*p* value
Confluence behind LA	6.8%	8.3%	0.825
No PV to LA seen	42.9%	44.0%	0.936
Abn coronary sinus	4.8%	2.4%	0.535
Abn Coumadin ridge	38.1%	20.2%	0.086
L : R disproportion	9.5%	2.4%	0.115
Abn 3VV spacing	9.5%	6.7%	0.613
Post-LA space	0.41 (SD 0.14)	0.31 (SD 0.09)	0.0001
Ao diameter	0.38 (SD 0.11)	0.32 (SD 0.08)	0.006
Post-LA index	1.19 (SD 0.61)	1.01 (SD 0.35)	0.081
Index >1.27	28.6%	17.9%	0.228

Legend. LA = left atrium. Abn = abnormal; L : R = left/right; 3VV = 3 vessel view; Ao = aorta.

**Table 2:** Sensitivity and specificity (with 95% confidence intervals) of the proposed diagnostic algorithms of TAPVR.

Diagnostic algorithm	Specificity (95% CI)	Sensitivity (95% CI)
Algorithm ^#^1—any 3+ markers	97.6% (91.5–99.7%)	10% (1.2–31.7%)
Algorithm ^#^2—any 4+ markers	98.8% (93.4–99.9%)	10% (1.2–31.7%)
Algorithm ^#^3—absence PVs & abn CR	90.5% (82.1–95.8%)	61.9% (38.3–61.9%)
Algorithm ^#^4—absence PVs & 3+ markers	94.1% (86.7–98.1%)	23.8% (8.2–47.2%)
Algorithm ^#^5—absence PVs & 4+ markers	95% (8.7%–98.1%)	18% (8.7–63%)

Legend. “absence of PVs & abn CR” (algorithm starts with stratification by nonvisualization of pulmonary vein entry into LA, followed by an abnormal Coumadin ridge); “absence of PVs & 3+ markers” (algorithm starts with stratification by nonvisualization of pulmonary vein entry into LA, followed by presence of 3 or more markers of TAPVR); “absence of PVs & 4+ markers” (algorithm starts with stratification by nonvisualization of pulmonary vein entry into LA, followed by presence of 4 or more markers of TAPVR).

## Data Availability

Data may be available upon reasonable request.
